# YOLO-LA: Prototype-Based Vision–Language Alignment for Silicon Wafer Defect Pattern Detection

**DOI:** 10.3390/mi17010067

**Published:** 2025-12-31

**Authors:** Ziyue Wang, Yichen Yang, Jianning Chu, Yikai Zang, Zhongdi She, Weikang Fang, Ruoxin Wang

**Affiliations:** 1 School of Intelligent Manufacturing, Jianghan University, Wuhan 430056, China; wangziyue0829@126.com; 2 Marine Design and Research Institute of China, Shanghai 200011, China; 3 Hubei Key Laboratory of Modern Manufacturing Quality Engineering, School of Mechanical Engineering, Hubei University of Technology, Wuhan 430068, China; 4 State Key Laboratory of Intelligent Manufacturing Equipment and Technology, School of Mechanical Science and Engineering, Huazhong University of Science and Technology, Wuhan 430074, China; 5 Department of Mechanical Engineering, University of Maryland College Park, College Park, MD 20742, USA

**Keywords:** semiconductor, defect detection, wafer bin map, YOLO, vision language model

## Abstract

With the rapid development of semiconductor manufacturing technology, methods to effectively control the production process, reduce variation in the manufacturing process, and improve the yield rate represent important competitive factors for wafer factories. Wafer bin maps, a method for characterizing wafer defect patterns, provide valuable information for engineers to quickly identify potential root causes through accurate pattern recognition. Vision-based deep learning approaches rely on visual patterns to achieve robust performance. However, they rarely exploit the rich semantic information embedded in defect descriptions, limiting interpretability and generalization. To address this gap, we propose YOLO-LA, a lightweight prototype-based vision–language alignment framework that integrates a pretrained frozen YOLO backbone with a frozen text encoder to enhance wafer defect recognition. A learnable projection head is introduced to map visual features into a shared embedding space, enabling classification through cosine similarity Experimental results on the WM-811K dataset demonstrate that YOLO-LA consistently improves classification accuracy across different backbones while introducing minimal additional parameters. In particular, YOLOv12 achieves the fastest speed while maintaining competitive accuracy, whereas YOLOv10 benefits most from semantic prototype alignment. The proposed framework is lightweight and suitable for real-time industrial wafer inspection systems.

## 1. Introduction

Semiconductor components require high manufacturing quality with minimal sur-face defects [1,2]. To overcome the brittleness and hardness of semiconductor materials, complex manufacturing processes have been proposed [3,4,5], and the intricacy of manufacturing defects in wafers produced during these processes has increased. A wafer bin map (WBM) [6] visually represents the test outcomes for each chip on a wafer, based on the chip’s probe test failure mode and its position (die). The chip probe test, an essential final assessment after the entire manufacturing sequence, evaluates the performance and functionality of each chip. During this test, each die is subjected to multiple probe test modes, with the first failure mode being recorded as the bin result. Throughout the wafer fabrication process, various manufacturing issues can lead to multiple defective dies on a wafer. These defects often cluster in specific areas on the wafer, forming spatial patterns known as gross failure areas (GFAs). These patterns, which include common types such as ring, scratch, loc, and center, are indicative of process-related issues and provide valuable data for improving yield and quality [7]. Classifying GFAs is crucial for engineers to identify and address problems in the production process, thereby reducing costs and enhancing yield. As production environments become more intricate, the need for automated WBM GFA classification has become increasingly important.

Deep learning-based object detection outperforms traditional algorithms and classification networks in terms of generalization ability and localization precision, leading to superior overall performance. Object detection algorithms can be categorized into two types: single-stage networks, exemplified by YOLO [8], SSD [9], and RetinaNet [10], and two-stage networks, represented by Faster R-CNN [11]. While two-stage networks prioritize accuracy over speed by separating localization and recognition, single-stage networks simultaneously perform both tasks, resulting in faster detection speeds. Although these vision-based models achieve competitive performance, they only rely on pixel-level information and ignore rich semantic knowledge inherent in defect pattern descriptions.

Meanwhile, recent progress in vision–language models (VLMs), such as CLIP-style architectures [12], demonstrates that aligning visual representations with natural-language semantics significantly improves generalization and interpretability. Much VLM research has been successfully applied in industrial defect detection. AnomalyGPT was proposed by Gu et al. [13] to address industrial anomaly detection by integrating fine-grained visual decoding and multi-turn dialog capability. It achieved state-of-the-art image- and pixel-level performance on MVTec-AD with strong few-shot generalization from only one normal example. Qian et al. [14] proposed a contrastive cross-modal training framework named CLAD that adopted large vision–language models to align visual and textual representations for industrial anomaly detection and localization. By jointly improving image-level detection and pixel-level localization on benchmarks such as MVTec-AD and VisA, it demonstrates that cross-modal contrastive learning can enhance both performance and interpretability in industrial inspection tasks. Cao et al. [15] developed a model named AnomalyVLM to address zero-shot industrial anomaly detection In AnomalyVLM, product standards were regarded as a substitute for reference images, enabling vision–language models to reason about normality and abnormality without category-specific training data.

However, integrating VLM concepts into industrial wafer map analysis remains largely unexplored. In particular, wafer defect classes naturally correspond to concise textual descriptions, suggesting an opportunity to leverage language as a source of domain knowledge. Therefore, in this paper, we introduce YOLO-LA, a prototype-based vision–language alignment framework, in which a trained YOLO backbone serves as a visual encoder, while a frozen text encoder generates semantic prototypes from defect descriptions. A lightweight learnable projection head aligns the YOLO visual embedding with the text embedding space, enabling wafer classification via cosine similarity to the textual prototypes.

## 2. YOLO Architectures and Baseline Model

YOLO, which stands for “You Only Look Once,” is a state-of-the-art, real-time object detection system that has revolutionized the field of computer vision. Developed by Joseph Redmon and his collaborators [8], YOLO is designed to detect and classify objects within images or video streams with remarkable speed and accuracy. Unlike traditional object detection methods that apply a classifier to various parts of an image, YOLO reframes object detection as a single regression problem, directly predicting bounding boxes and class probabilities from full images in one evaluation. This approach allows YOLO to process images in real time, making it highly efficient for applications that require fast and accurate object detection, such as autonomous driving, surveillance, and augmented reality. YOLO’s architecture is based on a convolutional neural network (CNN) that divides the input image into a grid and predicts bounding boxes and probabilities for each grid cell, enabling it to detect multiple objects of different classes simultaneously. Over the years, YOLO has undergone several iterations, each improving upon the previous in terms of speed, accuracy, and robustness, solidifying its position as a leading framework in the object detection domain.

### 2.1. YOLOv8

YOLOv8 was proposed by Jocher et al. [16]. In YOLOv8, the backbone is responsible for extracting hierarchical features from the input image. It consists of several sequential layers, including CBS, C2f, and SPPE. CBS typically stands for Convolution + BatchNorm + SiLU activation. It is used to process feature maps efficiently. C2f is a modified bottleneck structure, designed for feature extraction with fewer parameters. SPPF (Spatial Pyramid Pooling—Fast) enhances the receptive field and captures multi-scale features at the end of the backbone. The neck is designed to aggregate and fuse features from different stages of the backbone, helping detect objects at various scales. It includes C2f blocks for feature processing, Upsample operations to increase spatial resolution, and Concat operations to fuse feature maps from different layers. This part resembles a Feature Pyramid Network (FPN) combined with a Path Aggregation Network (PAN), enabling both top–down and bottom–up feature fusion. The head is responsible for final predictions.

### 2.2. YOLOv10

YOLOv10 [17] is the most representative model in YOLO series models. YOLOv10′s architecture leverages the strengths of its predecessors while incorporating several groundbreaking innovations. In contrast, in YOLOv10, the backbone utilizes an advanced version of C2F (cross-stage partial with two-fusion layers), called C2fCIB, which optimizes gradient flow and minimizes computational redundancy [18]. The neck module is designed to aggregate features from various scales, which employs PANet (Path Aggregation Network) layers to facilitate effective multi-scale feature fusion and pass them to the head.

### 2.3. YOLOv11

YOLOv11 [19] builds upon the foundation of YOLOv10, retaining its core design principles such as the modular backbone–neck–head architecture and the use of efficient components like CBS and SPPF blocks. Both versions adopt a one-to-many prediction strategy in the head, enabling each spatial location to generate multiple object detections. However, YOLOv11 introduces several key improvements over YOLOv10. YOLOv11 introduces a novel C3K2 (cross-stage partial with kernel size 2) module, which is an improved version of C2f, which combines the CSP concept with a smaller convolution kernel. By splitting the feature map into two parts, one is passed directly, while the other undergoes multiple bottleneck convolutions before being fused with the first part. This preserves rich features while reducing computation and parameter count, so as to improve computational efficiency and reduce inference latency. In addition, they also modified the PSA to C2PSA. In the C2PSA module, a convolutional layer with a kernel size of 1 is performed to split the feature map into two parts. One part enters the PSA module to highlight the response of key spatial regions, while the other part retains the original features. In the end, the two parts are concatenated and fused through another convolutional layer with a kernel size of 1.

### 2.4. YOLOv12

The YOLOv12 [20] architecture is composed of four key components, including a backbone module, neck module, and head module, where two techniques are applied to reduce computational cost. First, a residual efficient layer aggregation network (R-ELAN) in the backbone module was proposed to integrate features from different scales and reduce the computation cost and memory usage simultaneously. In addition, area attention was used in the neck module to reduce computation by narrowing the attention from global to local. The head part is the simple classification network, which uses a convolutional layer followed by an adaptive average pool layer. The last linear layer represents the output number of defect types.

## 3. YOLO-LA: Prototype-Based Vision–Language Alignment Framework

### 3.1. Overall Framework

**Figure 1:**
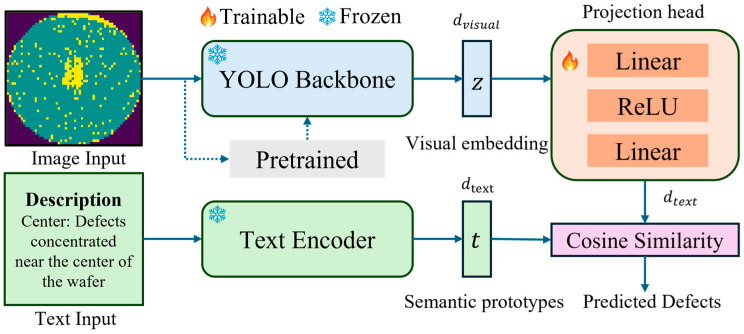
YOLO-LA framework. Given an input wafer map image, a pretrained YOLO backbone extracts visual features, which are mapped into the semantic embedding space via a lightweight projection head. Textual class descriptions are encoded into fixed semantic prototypes. Final classification is obtained by cosine similarity between visual embeddings and semantic prototypes. During training, the YOLO backbone is frozen unless otherwise specified.

A lightweight prototype-based vision–language alignment framework, termed YOLO-LA, is proposed, that enriches YOLO visual embeddings with semantic information extracted from natural-language defect descriptions [21]. Unlike standard CLIP pipelines that rely on large-scale image–text pretraining and open-vocabulary recognition, YOLO-LA operates on compact, topology-driven wafer maps and leverages frozen industrial visual backbones together with fixed semantic defect prototypes. YOLO-LA contains three major components, as shown in Figure 1: (1) a pretrained frozen YOLO backbone that produces visual embeddings of wafer maps, (2) a frozen text encoder that generates semantic prototypes from defect class descriptions, (3) a learnable projection head that aligns the visual and textual embedding spaces for prototype-based classification.

Visual Embedding Extraction: After training a YOLO classifier on wafer map dataset, the YOLO backbone is frozen and regarded as a visual feature extractor. Given an input image $x\in R^{H\times W\times 3}$, the YOLO backbone produces a high-level feature map $F\in R^{M\times S\times S}$ as Equation (1). By applying a global average pooling (*GAP*) layer, a compact visual descriptor $z\;{\in R}^{d_{visual}}$ is obtained for alignment.

(1) $$F=GAP(f_{yolo}\left(x\right))$$

(2) $$z=GAP\left(F\right)$$

Here, H and W  are the height and width of the input image, M and S are the number of channels and feature map size of the last feature layer of YOLO backbone, and $d_{visual}$ is the dimension of visual embedding.

Text Prototype Construction from Defect Descriptions: Each wafer defect class naturally corresponds to a short textual description. The defects and their descriptions are listed in Table 1. Each description is encoded to a text embedding $\;t_{c}\in R^{d_{text}}$ by a frozen sentence-level language model$\;f_{text}$ as Equation (3):

(3) $$t_{c}=f_{text}\left(d_{c}\right),\;c=\left\{1,2,\ldots C\right\}$$

where $d_{text}$ is the dimension of textual embedding, C is the number of defect types, and $d_{c}$ is a description of c-th defect type. Then, $l_{2}$ normalization is applied to obtain scaled text embeddings ${\tilde{t}}_{c}$. The final semantic prototype $t\;{\in R}^{{C\times d}_{text}}$ is generated by stacking all text embeddings as Equation (4) to serve as semantic anchors.

(4) $$t=\left[{\tilde{t}}_{1};{\tilde{t}}_{2},\ldots ,{\tilde{t}}_{C}\right]$$

**Projection Head for Visual–Language Alignment:** To align YOLO visual embedding z with language-based defect descriptions, a lightweight trainable two-layer MLP projection head is designed, as shown in Equations (5) and (6).

(5) $$h=Linear\left(ReLU\left(Linear\left(z\right)\right)\right)\in R^{d_{h}}$$

(6) $$v=Linear\left(h\right)\in R^{d_{text}}$$

Here, Linear and ReLU are the linear projection layer and activation function; $d_{h}$ is the dimension of the hidden layer; h and v are hidden embedding and aligned visual embedding. In this way, only the parameters of the projection head (<0.2 M parameters) are trained, while the YOLO backbone and text encoder remain frozen, ensuring high efficiency.

In the end, the final prediction is calculated by cosine similarity as Equation (7),

(7) $$\hat{y}=\arg\underset{c}{\max}{\tilde{v}}^{T}{\tilde{t}}_{c}$$

where $\tilde{v}$ is the $l_{2}$-normalized v.

### 3.2. Loss Function and Metrics

During training, a cross-entropy loss is utilized to optimize the projection head parameters as shown in Equation (8):

(8) $$L=-\sum_{i}log\frac{\exp\left({\tilde{v}}^{T}{\tilde{t}}_{c}\right)}{\sum_{c}\exp\left({\tilde{v}}^{T}{\tilde{t}}_{c}\right)}$$

In the paper, Accuracy [22] is adopted to evaluate the classification accuracy performance. Accuracy is defined as Equation (9):

(9) $$Accuracy=\frac{TP+TN}{TP+TN+FP+FN}$$

where TP is true positive, FN is false negative, TN is true negative, and FP is false positive. Top-N accuracy refers to the proportion of instances where the correct label is among the N predictions with the highest probabilities.

## 4. Experiments and Results

### 4.1. Dataset and Experimental Settings

**Figure 2:**
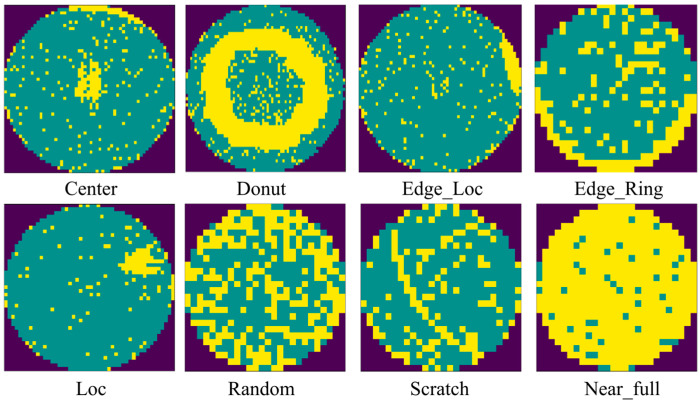
The eight types of defect pattern.

For training purposes, we utilized the WM-811K dataset [23], a comprehensive collection of 811,457 wafers sourced from real-world fabrication environments. The WM-811K dataset comprises 172,950 labeled samples and 639,507 unlabeled samples, with image sizes varying from 6 × 21 to 300 × 202 pixels. The labeled images are categorized into nine distinct types, including eight defect pattern types and a ‘None’ type representing normal wafers. In this study, we converted 25,519 labeled images with defect patterns into 3-channel images with a uniform size of 128 × 128 pixels. The dataset is first divided into training and test sets using an 80:20 stratified split. The training set is further split into training and validation subsets for model selection, while the test set is strictly held out for final evaluation. All splits are generated using a fixed random seed (42) and shared across all experiments. The eight defect types, including Center, Donut, Edge_Loc, Edge_Ring, Loc, Random, Scratch, and Near_full, are shown in Figure 2. In the pretraining process of the YOLO backbone, we set the training epoch as 50 and the optimizer as stochastic gradient descent with a learning rate of 0.01 and momentum of 0.9. The batch size is set as 16. All YOLO backbones used in this work are initialized with ImageNet-pretrained weights, following standard practice for classification tasks. The backbone networks are first trained using the native YOLO classification objective, which employs a SoftMax-based cross-entropy loss for multi-class classification. In the proposed YOLO-LA framework, the pretrained YOLO backbone is primarily used as a visual feature extractor. Unless otherwise specified in the ablation study, the backbone parameters are kept frozen, and only the lightweight projection head for semantic prototype alignment is optimized. This design ensures that the proposed method does not alter the original YOLO training objective but instead augments the learned visual representations through vision–language alignment. In YOLO-LA, MiniLM-L6-v2 is used for encoding each description, the training epoch is set to 100, the learning rate is 3 × 10^−4^, and the batch size and image size remain the same as in YOLO backbone pretraining. The $d_{h}$ in the projection head is 512. All experiments are run on a computer with an i7-9750H 2.60GHz CPU and NVIDIA GEFORCE RTX 2060 GPU.

### 4.2. Results

**Figure 3:**
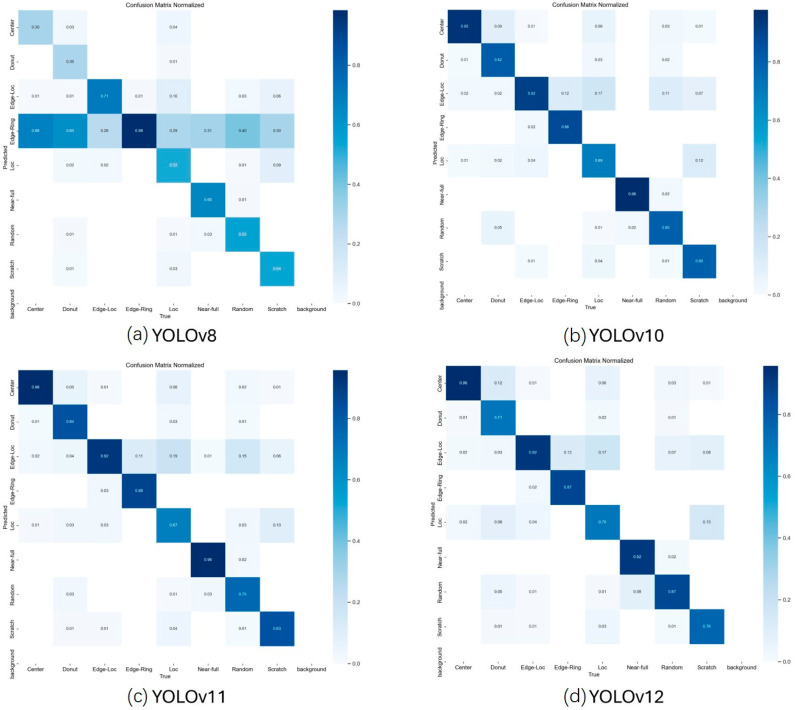
The confusion matrix of YOLOs.

First, we explore the YOLO backbone performance. Figure 3 shows confusion matrices. Figure 3a shows the result of YOLOv8, where the accuracy of some classes, such as Edge-Loc and Edge-Ring, was low, and there is significant misclassification of Random and Background. Many classes were less than 0.8, indicating relatively weak overall performance. Figure 3b demonstrates that YOLOv10 achieved accuracy between 0.85 and 0.99 in most defect types, significantly reducing confusion. In addition, background classification accuracy was near-perfect, slightly improving the overall accuracy. The results of YOLOv11 are shown in Figure 3c. The accuracy is high for most defect types, especially Near-full, Random, and Scratch, even approaching 1.0 for some types. However, defects with similar morphology, such as Edge_Loc and Edge_Ring, still exhibit confusion, although to a lesser extent than in YOLOv8 and YOLOv10. The results of YOLOv12 are shown in Figure 3d. The model achieves the best results on Center defect patterns, reaching 0.96, and the worst results on Loc defect patterns. The Loc defect pattern can be easily confused with Edge-Loc, and the Scratch defect pattern can be easily confused with Loc. The reason is that the YOLOv12 architectural design is consistent with more stable predictions on defect categories characterized by strong global spatial structures, such as Center and Edge_Ring, as reflected in the confusion matrix.

Then, we compared the YOLOv8-N, YOLOv10-N, YOLOv11-N, and YOLOv12-N architecture performance with the corresponding YOLO-LA model. As shown in Table 2, YOLO-LA can improve the YOLO performance whichever YOLO backbone is adopted. The YOLO-LA model based on the YOLOv10-N backbone network achieved the best performance, with the most significant improvement in semantic prototypes in YOLOv10-N, reaching 2.25%. YOLOv12-N achieved the fastest inference time (0.3 ms) and showed a competitive performance, also gaining +1.58%. The reason is that YOLOv12 includes enhanced operator fusion, reduced redundant computation, and more efficient feature aggregation, which collectively reduce inference latency. YOLOv11-N shows the smallest improvement (+0.39%). One reason is that the baseline YOLOv11 model already exhibits relatively balanced per-class performance, resulting in limited inter-class confusion and therefore less room for improvement through semantic alignment. These results indicate that semantic alignment should be viewed as a selective enhancement mechanism rather than a uniform performance booster, providing the greatest benefit for backbones and scenarios with higher baseline inter-class confusion. Overall, the proposed YOLO-LA framework is backbone-agnostic and provides consistent performance enhancement without modifying or fine-tuning the YOLO classifier itself.

**Figure 4:**
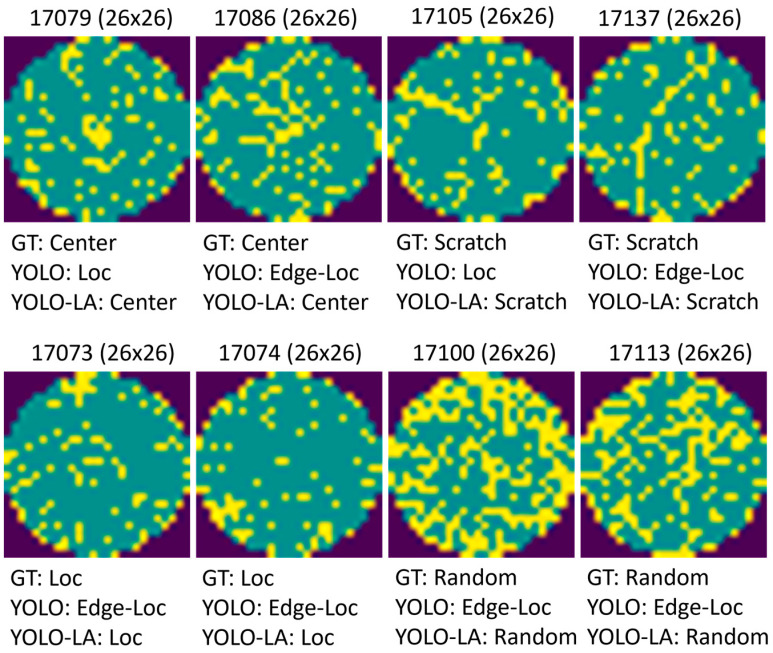
Visualization comparison results between YOLO and YOLO-LA.

In addition, Figure 4 presents representative classification examples comparing the baseline YOLO classifier and the proposed YOLO-LA model. Here, we used YOLOv10-N as the backbone. These cases show that YOLO-LA has a high ability to detect visually ambiguous defect patterns with high inter-class similarity. Specifically, the misclassified samples are all the extremely small effective resolution of wafer map images (approximately 26 × 26 pixels), which means purely visual models are prone to misinterpreting global structural patterns. YOLO-LA can introduce high-level structural priors from defect descriptions, leading to more robust classification under low-resolution conditions.

### 4.3. Ablation Study

Text Encoder. To further explore the performance of YOLO-LA, we conducted a series of text encoder studies using YOLOv10 backbones (Table 3), such as MiniLM-L3 [24], MiniLM-L6 [24], MiniLM-L12 [24], Mpnet-Base [25], Roberta-Large [26], and E5-Base [27]. The MiniLM family provides a favorable trade-off between efficiency and semantic representation quality. Among them, MiniLM-L12 showed the best classification performance. MiniLM-L6 with 33 M parameters achieved a balanced configuration and was therefore adopted as the default text encoder in most experiments. The text encoder serves as a semantic anchor for high-level attributes (e.g., concentration, locality) rather than a linguistic reasoning engine. In contrast, E5-base employed a larger backbone with higher-dimensional embeddings and achieved the second-best performance. However, the increased embedding dimension cannot ensure better performance and slightly increases training costs. This indicates that the effectiveness of YOLO-LA does not rely on the expressive power of large language models but instead on introducing structured semantic priors into the visual decision process.

Prompt Type: As shown in Table 4, we compared different prompt types, including base, detailed, and concise descriptions of defects. The detailed and concise descriptions for each defect are listed in Appendix A Table A1 and Table A2. It is observed that the base prompt achieved the best overall performance, with an accuracy of 84.44% and a macro-F1 of 51.16%, while also requiring the shortest training time. Macro-F1 is adopted to evaluate per-class performance by equally weighting the F1 score of each defect category. This suggests that semantic prototype alignment benefits mainly from high-level structural semantics rather than prompt verbosity and that both overly detailed and overly compressed descriptions can introduce redundant or ambiguous cues. Based on the overall accuracy, it can be concluded that this process is not dominated by specific keywords within the embeddings.

Training Strategy: We further explored the effect of different training strategies, including freezing the YOLO backbone, partially fine-tuning the last two blocks, and fully fine-tuning the entire network. As shown in Table 5, freezing the backbone consistently yields the best performance, achieving 85.31% accuracy and 51.88% macro-F1, while also being the most computationally efficient. In contrast, partial and full fine-tuning lead to longer training times and noticeable performance degradation. This indicates that, under low-resolution wafer map settings, aggressive fine-tuning may disturb pretrained visual representations and increase overfitting risk.

Projection Head: The projection head architecture was further ablated in terms of activation function, hidden dimension, and network depth. The results in Table 6 and Table 7 show that increasing the hidden dimension from 256 to 512 consistently improves both accuracy and macro-F1, while larger depths bring marginal or unstable gains. Among different activation functions, GELU slightly outperforms ReLU for smaller hidden dimensions, whereas their performance becomes comparable at higher dimensions. Overall, a two-layer projection head with 512 hidden units provides the best balance between performance and training efficiency, supporting the use of a lightweight alignment module.

YOLO Backbone: To explore the performance of YOLOs, we compared the performance of different YOLOs architectures. The detailed results are shown in Table 8, Table 9, Table 10 and Table 11. There are clear differences in terms of design objectives, computational efficiency, and accuracy. YOLOv8 showed a steady scaling trend in parameters with training time increasing from 6.9 h to 58 h and inference latency from 2.0 ms to 29.2 ms. While providing balanced performance, its accuracy remains relatively stable between 0.833 and 0.839, showing limited improvement with model size, indicating that for topology-driven task, excessive model capacity may amplify sensitivity to noise and local irregularities. These observations highlight that semantic prototype alignment acts as a form of semantic regularization, the benefit of which depends on the balance between visual representation capacity and task characteristics. YOLOv10 showed high accuracy enhancement, achieving the highest accuracy among the four YOLO versions, with up to 0.853 in the YOLO with s size. However, this improvement comes with the downside of significantly deeper networks, longer training times, and slower inference. Therefore, YOLOv10 is more suitable for scenarios where maximum accuracy is required and computational resources are sufficient. YOLOv11 shows a more balanced trade-off between accuracy and efficiency. With Top-1 accuracy in the range of 0.84–0.851, it remains competitive with YOLOv10 while reducing both training and inference overheads. YOLOv12 represents a breakthrough in computational efficiency. It maintains a compact parameter size while dramatically reducing the training time and inference latency. In summary, YOLOv10 achieved superior accuracy at the expense of computational cost, YOLOv11 achieved a more practical balance between accuracy and efficiency, and YOLOv12 offered a substantial leap in training and inference efficiency with minimal accuracy loss, making it particularly attractive for real-time and resource-constrained applications.

## 5. Conclusions

In this paper, we propose YOLO-LA, a lightweight prototype-based vision–language alignment framework that integrates a frozen YOLO classifier backbone with a frozen text encoder for wafer pattern detection. Semantic text prototypes are generated from natural-language defect descriptions using a text encoder, enabling interpretable classification via cosine similarity in a shared embedding space. In addition, an efficient learnable projection head is designed to achieve minimal extra computation. Extensive experiments on wafer map defect classification demonstrate that semantic alignment consistently improves classification robustness, particularly under low-resolution conditions where purely visual cues are insufficient to distinguish structurally similar defect patterns. The results further show that the proposed framework is robust to prompt formulation, benefits from a frozen visual backbone, and achieves an effective performance–efficiency trade-off with a lightweight projection head. In future work, we will explore few-shot and zero-shot defect recognition through dynamic prototype updating, as well as validation on real-world production data from semiconductor fabrication lines.

## Figures and Tables

**Table 1:** Textual description of defects.

Defect Type	Description
Center	Defects concentrated near the center of the wafer
Donut	Donut-shaped defect pattern on the wafer
Edge_Ring	Ring-shaped defects along the wafer edge
Edge_Loc	Localized defects near the wafer edge
Loc	Localized defect cluster in a small region
Random	Randomly scattered defects on the wafer
Scratch	Linear scratch-like defects across the wafer
Near_full	Defects covering most of the wafer area

**Table 2:** The performance comparison of different YOLOs. Bold numbers indicate the best performance for each column, and underlined numbers indicate the second-best performance.

Backbone	Params (M)	Inference Time (ms)	Accuracy % (YOLO)	Accuracy % (YOLO-LA)
YOLOv8-N	1.4	3.0	83.28	84.85 (+1.54)
YOLOv10-N	0.9	6.1	83.50	**85.75** (**+2.25**)
YOLOv11-N	1.5	1.3	**83.61**	84.00 (+0.39)
YOLOv12-N	1.7	**0.3**	83.48	85.06 (+1.58)

**Table 3:** The ablation study for various text encoders. Bold numbers indicate the best performance for each column, and underlined numbers indicate the second-best performance.

Text Encoder	$d_{text}$	Params (M)	Encoding Time (ms)	Accuracy (%)
MiniLM-L3	384	22	**18.72**	85.72
MiniLM-L6	384	33	31.88	85.75
MiniLM-L12	384	66	60.28	**85.90**
Mpnet-Base	768	110	59.79	85.67
RoBERTa-Large	1024	355	83.58	85.77
E5-base	768	110	46.68	85.88

**Table 4:** The ablation studies for various prompt types.

Prompt Type	Training Time (s)	Accuracy (%)	Macro-F1 (%)
Base	327.30	84.44	51.16
Detailed	405.11	83.26	50.37
Concise	405.97	82.11	49.87

**Table 5:** The ablation studies for different training strategies.

Training Strategy	Training Time (s)	Accuracy (%)	Macro-F1 (%)
Freeze	418.42	85.31	51.88
Last-2	464.03	83.31	50.38
All	564.45	79.42	47.96

**Table 6:** The ablation study for different activation functions and hidden dimensions.

Activation Function	Dimensions	Training Time (s)	Accuracy (%)	Macro-F1 (%)
Relu	256	402.84	82.59	49.86
Gelu	256	405.33	84.52	51.37
Relu	512	419.35	84.82	51.36
Gelu	512	417.25	83.87	51.01

**Table 7:** The ablation study for different hidden layers.

Hidden Layer	Training Time (s)	Accuracy (%)	Macro-F1 (%)
1	394.37	83.59	50.54
2	419.35	84.82	51.36
3	429.29	84.05	51.39

**Table 8:** The performance of different YOLOv8 architectures.

YOLOv8 Size	N	S	M	L	X
Params (M)	1.4	5.1	15.8	36.2	56.1
Training Time (h)	6.9	10.7	31.8	40.4	58.0
Inference Time (ms)	2.0	4.3	9.7	20.3	29.2
Layers	73	73	103	133	133
Accuracy	0.839	0.835	0.836	0.835	0.833

**Table 9:** The performance of different YOLOv10 architectures.

YOLOv10 Size	N	S	M	L	X
Params (M)	0.9	2.9	8.3	20.2	30.1
Training Time (h)	7.4	13.9	28.9	55.5	73.15
Inference Time (ms)	2.8	6.1	13.4	28.1	36.5
Layers	89	126	185	244	244
Accuracy	0.844	0.853	0.845	0.852	0.85

**Table 10:** The performance of different YOLOv11 architectures.

YOLOv11 Size	N	S	M	L	X
Params (M)	1.5	5.4	10.4	12.8	28.3
Training Time (h)	8.2	13.0	27.4	32.1	54.2
Inference Time (ms)	2.4	5.3	14.1	15.5	28.9
Layers	112	112	138	227	227
Accuracy	0.84	0.848	0.844	0.85	0.851

**Table 11:** The performance of different YOLOv12 architectures.

YOLOv12 Size	N	S	M	L	X
Params (M)	1.7	6.1	12.0	15.4	34.0
Training Time (h)	1.5	1.7	2.2	3.256	9.25
Inference Time (ms)	0.3	0.4	0.6	0.7	1.2
Layers	94	94	104	190	190
Accuracy	0.842	0.837	0.844	0.845	0.843

## Data Availability

The original contributions presented in the study are included in the article; further inquiries can be directed to the corresponding author.

## Appendix A

**Table A1:** Textual detailed description of defects.

Defect Type	Description
Center	Most defective dies are densely concentrated around the wafer center (central cluster).
Donut	Defects form a donut pattern: a ring-like distribution with a relatively cleaner center.
Edge_Ring	Defects are distributed along the wafer perimeter, forming a ring-like band near the edge.
Edge_Loc	Defects form a localized cluster near the wafer boundary/edge, not forming a full ring.
Loc	Defects are confined to a small local region (single compact cluster) inside the wafer.
Random	Defects are scattered randomly across the wafer with no clear spatial structure.
Scratch	Defects align into a long, thin, linear streak crossing the wafer (scratch-like line).
Near_full	Defects cover most of the wafer surface area (nearly full wafer affected).

**Table A2:** Textual concise description of defects.

Defect Type	Description
Center	Center cluster
Donut	Donut ring
Edge_Ring	Edge ring band
Edge_Loc	Edge local cluster
Loc	Local cluster
Random	Random scatter
Scratch	Linear scratch
Near_full	Nearly full coverage
